# Complete Genome Sequences of Eight *Faecalibacterium* sp. Strains Isolated from Healthy Human Stool

**DOI:** 10.1128/mra.00824-22

**Published:** 2022-12-13

**Authors:** Davide Fraccascia, Ryan M. Chanyi, Eric Altermann, Nicole C. Roy, Steve H. Flint, Warren C. McNabb

**Affiliations:** a Riddet Institute, Massey University, Palmerston North, New Zealand; b AgResearch, Grasslands Research Centre, Palmerston North, New Zealand; c School of Food and Advanced Technology, Massey University, Palmerston North, New Zealand; d High-Value Nutrition National Science Challenge, Auckland, New Zealand; e Department of Human Nutrition, University of Otago, Dunedin, New Zealand; f School of Veterinary Science, Massey University, Palmerston North, New Zealand; University of Maryland School of Medicine

## Abstract

Eight *Faecalibacterium* sp. strains were isolated from feces of healthy human volunteers. Here, we describe their genome sequences. The genome sizes ranged from 2.78 Mbp to 3.23 Mbp, with an average GC content of 56.6% and encoding 2,795 protein-coding genes on average.

## ANNOUNCEMENT

*Faecalibacterium* sp. are commensal microorganisms found ubiquitously in the human gastrointestinal tract (GIT). These microbes are important species contributing to human health through the production of butyrate, which is thought to have health-promoting properties. A reduction in *Faecalibacterium* in patients with different forms of inflammatory bowel disease has led researchers to believe these microorganisms confer health benefits (1–7).

This study isolated and sequenced eight strains of *Faecalibacterium* from human fecal samples collected in Palmerston North, New Zealand. Donors were recruited according to Massey University Ethics Approval (SOA 19/03). Volunteers were deemed healthy if they had a body mass index between 18.5 and 30; had no history of antibiotics, laxatives, or GIT infections or disorders 3 months prior to sample collection; and had moderate fiber consumption (>15 g/day). Samples were collected and processed as described by Fitzgerald et al. (8) using yeast casitone fatty acid medium supplied with glucose (YCFAG). Strain HTF-F (9) was also sequenced for comparison as a strain of interest due to its unique extrapolymeric matrix (2).

To isolate DNA, bacteria were cultured in YCFAG at 37°C overnight in an anaerobic workstation (75% N_2_, 15% CO_2_, and 5% H_2_; DonWhitley Scientific, UK). Samples were concentrated via centrifugation at 8,000 × *g* and processed using the Nucleospin soil genomic DNA purification kit (Macherey-Nagel) as per the manufacturer’s protocol. Library preparation and sequencing, including quality control (QC), was handled by Massey Genome Service (MGS; Massey University, New Zealand), using the Illumina Nextera XT kit on the Illumina MiSeq 2 × 300-bp paired-end (PE) v3 platform. Each sequence was trimmed to their longest contiguous segment within a quality cutoff (0.01), using the dynamictrim application from the SolexaQA++ software (v3.1.7.2; http://solexaqa.sourceforge.net/). Quality checking was conducted using standard parameters with FastQC (v0.11.9) (10).

For long-read sequencing, bacteria were grown again as described above, and DNA was extracted using Qiagen Genomic-tip 100/G columns per the manufacturer’s protocol. Mutanolysin (100 U; Sigma-Aldrich) and MetaPolyzyme (10 μL/sample; Sigma-Aldrich) were added to enhance bacterial lysis. Samples were sent to MGS for sample quality assessment and to Novogene (Singapore) for PacBio sequencing.

PacBio sequencing, including library preparation and QC, were performed by Novogene. The PacBio SMRTbell library was created by shearing template DNA, and the hairpin-legated dimers were purified by magnetic beads with 10-kilonucleotide size selection conditions. The library was checked with Qubit and Bioanalyzer for quantification and size distribution, respectively. Quantified libraries were pooled and sequenced on PacBio Sequel II/IIe system. The PacBio subreads and *N*_50_ values are listed in Table 1.

**TABLE 1:** List of *Faecalibacterium* sp. strain information from this study

Strain	Illumina read count (paired)	PacBio reads *N*_50_ (bp)	No. of PacBio subreads	GC content (%)	No. of DNA CDS^*a*^	No. of rRNAs	No. of tRNAs	Coding ratio (%)	Length (bp)	Accession no. of:			
										BioSample	Genomes	Assembly	SRA
IP-1-18	543,181	14,826	101,178	56.2	2,814	18	64	86.2	3,038,545	SAMN26934697	NZ_CP094472.1	GCA_023347355.1	SRX15120859, SRX15120845
IP-3-29	667,687	12,497	387,776	56.9	2,748	18	65	86.7	3,002,063	SAMN26934698	NZ_CP094471.1	GCA_023347335.1	SRX15120860, SRX15120846
I2-3-92	704,052	13,398	279,899	56.8	2,774	18	68	87.1	2,963,404	SAMN26934699	NZ_CP094470.1	GCA_023347315.1	SRX15120853, SRX15120847
HTF-F	794,651	14,932	160,344	56.6	2,571	18	65	85.7	2,776,287	SAMN26934700	NZ_CP094473.1	GCA_023347535.1	SRX15120854, SRX15120848
I3-3-33	596,779	14,126	491,692	56.6	2,669	18	65	85.4	2,994,777	SAMN26934701	NZ_CP094469.1	GCA_023347295.1	SRX15120855, SRX15120849
I3-3-89	586,081	10,975	300,107	58	2,698	18	65	86	2,816,187	SAMN26934702	NZ_CP094468.1	GCA_023347275.1	SRX15120856, SRX15120850
I4-1-79	650,123	15,580	195,760	56	3,101	18	67	85.7	3,227,950	SAMN26934703	NZ_CP094467.1	GCA_023347235.1	SRX15120857, SRX15120851
I4-3-84	576,265	15,227	367,152	55.5	2,984	18	70	86.2	3,119,411	SAMN26934704	NZ_CP094466.1	GCA_023347255.1	SRX15120858, SRX15120852

a CDS, coding DNA sequences.

Raw PacBio reads were filtered via Filtlong (https://github.com/rrwick/Filtlong) using a minimum subread length of 1,000 bases and a 95% cutoff. High-coverage long reads were assembled using Trycycler v0.5.3 (11), Miniasm v0.3-r179 (12), and Flye v2.9-b1768 (13) and polished with Polypolish v0.5.0 (14). Strains with low-coverage long-read data were combined with their Illumina data and hybrid assembled using Unicycler (v0.5) (12). Assembly integrity was assessed (QUAST and CheckM) on the online platform Kbase (https://kbase.us). Default parameters for all software were used. Genome annotation was performed using the NCBI Prokaryotic Genome Annotation Pipeline (PGAP) (Table 1) (15–17). Trycycler and Unicycler confirmed all genomes to be circular.

### Data availability.

All the annotated genomes and the respective long and short raw reads have been deposited in GenBank under BioProject PRJNA819544. Assembly, BioSample, and SRA details are specified in Table 1.

## References

[1] Miquel S, Martín R, Lashermes A, Gillet M, Meleine M, Gelot A, Eschalier A, Ardid D, Bermúdez-Humarán LG, Sokol H, Thomas M, Theodorou V, Langella PM, Carvalho FA. 2016. Anti-nociceptive effect of Faecalibacterium prausnitzii in non-inflammatory IBS-like models. Sci Rep 6:19399. doi:10.1038/srep19399. 26775847 PMC4726104

[2] Rossi O, Khan MT, Schwarzer M, Hudcovic T, Srutkova D, Duncan SH, Stolte EH, Kozakova H, Flint HJ, Samsom JN, Harmsen HJM, Wells JM. 2015. Faecalibacterium prausnitzii strain HTF-F and its extracellular polymeric matrix attenuate clinical parameters in DSS-induced colitis. PLoS One 10:e0123013. doi:10.1371/journal.pone.0123013. 25910186 PMC4409148

[3] Laval L, Martin R, Natividad JNF, Chain FC, Miquel S, Desclée de Maredsous C, Capronnier S, Sokol H, Verdu EF, van Hylckama Vlieg JET, Bermúdez-Humáran LG, Smokvina T, Langella P, Chain F, Miquel S, Desclée de Maredsous C, Capronnier S, Sokol H, VerdU EF, van Hylckama Vlieg JET, Bermúdez-Humarán LG, Smokvina T, Langella P. 2015. Lactobacillus rhamnosus CNCM I-3690 and the commensal bacterium faecalibacterium prausnitzii A2-165 exhibit similar protective effects to induced barrier hyper-permeability in mice. Gut Microbes 6:1–9. doi:10.4161/19490976.2014.990784. 25517879 PMC4615674

[4] Martín R, Miquel S, Chain F, Natividad JM, Jury J, Lu J, Sokol H, Theodorou V, Bercik P, Verdu EF, Langella P, Bermúdez-Humarán LG. 2015. Faecalibacterium prausnitzii prevents physiological damages in a chronic low-grade inflammation murine model. BMC Microbiol 15:67. doi:10.1186/s12866-015-0400-1. 25888448 PMC4391109

[5] Quévrain E, Maubert MA, Michon C, Chain F, Marquant R, Tailhades J, Miquel S, Carlier L, Bermúdez-Humarán LG, Pigneur B, Lequin O, Kharrat P, Thomas G, Rainteau D, Aubry C, Breyner N, Afonso C, Lavielle S, Grill J-P, Chassaing G, Chatel JM, Trugnan G, Xavier R, Langella PM, Sokol H, Seksik P. 2016. Identification of an anti-inflammatory protein from Faecalibacterium prausnitzii, a commensal bacterium deficient in Crohn’s disease. Gut 65:415–425. doi:10.1136/gutjnl-2014-307649. 26045134 PMC5136800

[6] Bridonneau C, Altare F, Tabiasco J, Alameddine J, Godefroy E, Sokol H, Yazdanbakhsh K, Papargyris L, Sarrabayrouse G, Jotereau F. 2019. Faecalibacterium prausnitzii Skews Human DC to Prime IL10-Producing T Cells Through TLR2/6/JNK Signaling and IL-10, IL-27, CD39, and IDO-1 Induction. Front Immunol 10:143. doi:10.3389/fimmu.2019.00143. 30787928 PMC6373781

[7] Hu W, Lu W, Li L, Zhang H, Lee Y, Chen W, Zhao J. 2021. Both living and dead Faecalibacterium prausnitzii alleviate house dust mite-induced allergic asthma through the modulation of gut microbiota and short-chain fatty acid production. J Sci Food Agric 101:5563–5573. doi:10.1002/jsfa.11207. 33709404

[8] Fitzgerald CB, Shkoporov AN, Sutton TDS, Chaplin AV, Velayudhan V, Ross RP, Hill C. 2018. Comparative analysis of Faecalibacterium prausnitzii genomes shows a high level of genome plasticity and warrants separation into new species-level taxa. BMC Genomics 19:931. doi:10.1186/s12864-018-5313-6. 30547746 PMC6295017

[9] Lopez-Siles M, Khan TM, Duncan SH, Harmsen HJM, Garcia-Gil LJ, Flint HJ. 2012. Cultured representatives of two major phylogroups of human colonic Faecalibacterium prausnitzii can utilize pectin, uronic acids, and host-derived substrates for growth. Appl Environ Microbiol 78:420–428. doi:10.1128/AEM.06858-11. 22101049 PMC3255724

[10] Andrews S. 2010. FastQC: a quality control tool for high throughput sequence data. https://www.bioinformatics.babraham.ac.uk/projects/fastqc/.

[11] Wick RR, Judd LM, Cerdeira LT, Hawkey J, Méric G, Vezina B, Wyres KL, Holt KE. 2021. Trycycler: consensus long-read assemblies for bacterial genomes. Genome Biol 22:266. doi:10.1186/s13059-021-02483-z. 34521459 PMC8442456

[12] Wick RR, Judd LM, Gorrie CL, Holt KE. 2017. Unicycler: resolving bacterial genome assemblies from short and long sequencing reads. PLoS Comput Biol 13:e1005595. doi:10.1371/journal.pcbi.1005595. 28594827 PMC5481147

[13] Kolmogorov M, Yuan J, Lin Y, Pevzner PA. 2019. Assembly of long, error-prone reads using repeat graphs. Nat Biotechnol 37:540–546. doi:10.1038/s41587-019-0072-8. 30936562

[14] Wick RR, Holt KE. 2022. Polypolish: short-read polishing of long-read bacterial genome assemblies. PLoS Comput Biol 18:e1009802. doi:10.1371/journal.pcbi.1009802. 35073327 PMC8812927

[15] Haft DH, DiCuccio M, Badretdin A, Brover V, Chetvernin V, O'Neill K, Li W, Chitsaz F, Derbyshire MK, Gonzales NR, Gwadz M, Lu F, Marchler GH, Song JS, Thanki N, Yamashita RA, Zheng C, Thibaud-Nissen F, Geer LY, Marchler-Bauer A, Pruitt KD. 2018. RefSeq: an update on prokaryotic genome annotation and curation. Nucleic Acids Res 46:D851–D860. doi:10.1093/nar/gkx1068. 29112715 PMC5753331

[16] Li W, O'Neill KR, Haft DH, DiCuccio M, Chetvernin V, Badretdin A, Coulouris G, Chitsaz F, Derbyshire MK, Durkin AS, Gonzales NR, Gwadz M, Lanczycki CJ, Song JS, Thanki N, Wang J, Yamashita RA, Yang M, Zheng C, Marchler-Bauer A, Thibaud-Nissen F. 2021. RefSeq: expanding the Prokaryotic Genome Annotation Pipeline reach with protein family model curation. Nucleic Acids Res 49:D1020–D1028. doi:10.1093/nar/gkaa1105. 33270901 PMC7779008

[17] Tatusova T, Dicuccio M, Badretdin A, Chetvernin V, Nawrocki EP, Zaslavsky L, Lomsadze A, Pruitt KD, Borodovsky M, Ostell J. 2016. NCBI Prokaryotic Genome Annotation Pipeline. Nucleic Acids Res 44:6614–6624. doi:10.1093/nar/gkw569. 27342282 PMC5001611

